# Preferences for Mobile-Supported e-Cigarette Cessation Interventions Among Young Adults: Qualitative Descriptive Study

**DOI:** 10.2196/33640

**Published:** 2022-04-01

**Authors:** Zil E Huma, Laura Struik, Joan L Bottorff, Mohammad Khalad Hasan

**Affiliations:** 1 School of Nursing University of British Columbia Kelowna, BC Canada

**Keywords:** qualitative research, electronic nicotine delivery systems, e-cigarette, cessation, young adults, smartphone apps, mHealth, mobile phone

## Abstract

**Background:**

Despite the steady rise in electronic cigarette (e-cigarette) uptake among young adults, increasingly more young people want to quit. Given the popularity of smartphones among young adults, mobile-based e-cigarette cessation interventions hold significant promise. Smartphone apps are particularly promising due to their varied and complex capabilities to engage end users. However, evidence around young adults’ preferences and expectations from an e-cigarette cessation smartphone app remains unexplored.

**Objective:**

The purpose of this study was to take an initial step toward understanding young adults’ preferences and perceptions on app-based e-cigarette cessation interventions.

**Methods:**

Using a qualitative descriptive approach, we interviewed 12 young adults who used e-cigarettes and wanted to quit. We inductively derived themes using the framework analysis approach and NVivo 12 qualitative data analysis software.

**Results:**

All participants agreed that a smartphone app for supporting cessation was desirable. In addition, we found 4 key themes related to their preferences for app components: (1) flexible personalization (being able to enter and modify goals); (2) e-cigarette behavior tracking (progress and benefits of quitting); (3) safely managed social support (moderated and anonymous); and (4) positively framed notifications (encouraging and motivational messages). Some gender-based differences indicate that women were more likely to use e-cigarettes to cope with stress, preferred more aesthetic tailoring in the app, and were less likely to quit cold turkey compared with men.

**Conclusions:**

The findings provide direction for the development and testing of app-based e-cigarette cessation interventions for young adults.

## Introduction

Electronic cigarettes, or e-cigarettes, deliver nicotine via inhaled vapor [1], which is why e-cigarette use is often called “vaping.” The use of e-cigarettes has risen exponentially in North America, especially among young adults. According to the Canadian Tobacco and Nicotine Survey conducted between December 2020 and January 2021 [2], 15% of those aged 20-24 reported e-cigarette use in the past month and 48% reported having tried e-cigarettes once at some point. Similarly, in the United States, there has been a surge of nicotine-based e-cigarette uptake among young adults, with 20% of Americans aged 18-29 using e-cigarette products [3]. Uptake of e-cigarettes among young adults is occurring among both smokers and nonsmokers, with curiosity (27%) and smoking cessation (20%) the primary reasons for uptake among 20-24 year olds, and smoking cessation (41%) and avoidance of cigarettes (17%) the primary reasons for uptake among those 25 and older in Canada [4].

Regardless of the reason for e-cigarette uptake, an increasing number of young people have indicated a desire to quit. Between 2018 and 2019, Canadian young adults who used e-cigarettes indicated that they were planning to quit in the next 6 months [5]. According to a recent study by Sanchez et al [6], 44% of Canadian young adults between 18 and 29 years of age had tried to quit, 33% were thinking about quitting, and 15% made plans to quit e-cigarettes. In the United States, 62.4% of current adult e-cigarette users, with the majority consisting of young adults (ages 18-34 years), planned to quit e-cigarettes for good, and 25% reported a past-year quit attempt [7]. Moreover, a similar study conducted by Berg et al [8] found that 20.8% of young adult e-cigarette users reported their willingness to quit e-cigarettes in the next 6 months and 32.3% reported past-year quit attempts.

Providing interventions to help support reduction and cessation of nicotine dependence associated with e-cigarettes is a promising way to help young adults in their cessation journey. e-Cigarette cessation support is in a nascent stage of development and to date, includes in-person counseling, group-based counseling, and quit lines [9]. Given the popularity of smartphones among young adults [10], a few mobile-based interventions are now on the market. One of them includes an SMS text messaging intervention to promote e-cigarette cessation among young adults [11], for which preliminary evidence in relation to user engagement and effectiveness is promising [11,12].

Smartphone apps provide promising ways to engage young adults in behavior change interventions, including the opportunity to incorporate gamification [13-15]. Not surprisingly, smartphone apps for e-cigarette cessation have received widespread support among young adults [8,16]. However, evidence around young adults’ preferences and expectations from an e-cigarette cessation app remains unexplored. Therefore, in this study, we took an initial step toward understanding young adults’ preferences and perceptions on app-based e-cigarette cessation interventions.

## Methods

### Design

We used a qualitative descriptive study approach. Qualitative description is appropriate because it aims to help researchers explore and understand end user perspectives or experiences on a topic where little is known [17,18]. This aligns with the goal of this study, which is to provide a rich description of young adults’ perspectives and preferences for an app-based e-cigarette cessation support.

### Ethics Approval

Ethics approval for this study was obtained from the University of British Columbia (Okanagan campus) Behavioral Research Ethics Board (BREB #: H21-00272).

### Participants

We purposively recruited 12 young adults’ aged 20-29 (mean age 25.5 [SD 2.66] years) who were current e-cigarette users and wanted to quit or reduce the use of e-cigarettes. The recruitment procedure included a single online advertisement that was distributed across 3 platforms (Kijiji [19], Castanet [20], and university web postings) with the caption “If you vape and want to quit, we want to hear from you!” All participants were recruited within 2 weeks of releasing the online recruitment advertisements. All participants provided informed consent prior to data collection.

### Data Collection

Data collection included semistructured interviews conducted online using Zoom (Zoom Video Communications, Inc.). At the start of the interview, participants were asked to answer a short questionnaire to gather demographic (eg, age, gender) and e-cigarette information (eg, e-cigarette usage history, quit attempts, app use). For each interview, the same interview guide was followed. During the interviews, participants reported on their perceptions and preferences for receiving app-based support for reducing and quitting e-cigarettes, with interview prompts centered on common behavior change app features (eg, personalization, behavior tracking, social support, and notifications). The interviews were audio recorded and lasted approximately 45 minutes each. Each participant received a CAD $40 (US $32) e-gift card for their participation. The interview questions are presented in Multimedia Appendix 1.

### Data Analysis

We adopted the framework analysis approach [21] to inductively develop themes within the qualitative data. The framework approach is a flexible and systematic approach that is ideal for developing themes from semistructured interviews [22]. This approach is hallmarked by a series of clear stages so that a coherent audit trail is provided during thematic development [22]. This process of thematic development allowed us to compare and contrast findings, particularly to assess gender influences.

The interviews were first transcribed verbatim and uploaded in the qualitative data analysis software program NVivo version 12 (QSR International) [23]. We disaggregated the data by gender to identify notable differences in e-cigarette patterns as well as needs and preferences related to e-cigarette cessation. After a detailed reading of the transcripts in their entirety to become familiar with the data, 2 researchers (ZH and LS) iteratively developed a coding framework using data from the interviews with young women and young men. All authors then reviewed and approved the framework. One author (ZH) then coded major themes and subsequent subthemes in relation to the analytical framework in NVivo, which were revised or added to as new data were collected.

## Results

### Sample Characteristics

We interviewed 12 e-cigarette users who were interested in quitting. The mean age of participants was 25.5 (ranging from 19 to 29) years, 50% (6/12) were male, 50% (6/12) were Caucasian, and most (9/12, 75%) completed postsecondary education (diploma or degree), and worked either part-time or full-time (7/12, 58%; Table 1). Most participants vaped more than once/day (9/12, 75%), having been using e-cigarettes for 6 months to a year (7/12, 58%), and primarily used devices with refillable cartridges (9/12, 75%). Men mostly reported e-cigarette uptake to replace smoking, while women also reported e-cigarette uptake to manage stress, or due to peer influence. Most participants reported wanting to quit for the last 6 months (7/12, 58%), and have made more than 3 quit attempts (8/12, 67%). Health and money were the primary reasons for quitting. While more men preferred to quit cold turkey compared with women, an equal number also preferred gradual reduction. None of the participants had used an app to help them quit e-cigarettes.

**Table 1:** Participant demographic and e-cigarette^a^ information.

Demographic and e-cigarette information		Male (n=6)	Female (n=6)		Total (n=12)		
**Education**							
	Some postsecondary	1		2		3	
	Certificate/diploma	3		2		5	
	University degree	2		2		4	
**Employment**							
	Full-time	3		2		5	
	Part-time	1		1		2	
	Other (student, unemployed, caregiver)	2		3		5	
**Ethnicity**							
	White/Caucasian	4		2		6	
	Asian	1		2		3	
	Black/African American	1		2		3	
	White/East Indian	0		1		1	
**Reasons for e-cigarette**							
	Smoking alternative	5		2		7	
	Something new	1		0		1	
	Manage stress	0		2		2	
	Peer influence	0		2		2	
**Frequency of e-cigarette**							
	More than once/day	5		4		9	
	About once/day	1		0		1	
	A few times/week	0		2		2	
**Preferred vape device**							
	Device with prefilled cartridges	6		3		9	
	Device that allows user to fill	0		1		1	
	Disposable	0		2		2	
**History of e-cigarette**							
	More than a year	3		1		4	
	6 months to 1 year	3		4		7	
	Less than 6 months	0		1		1	
**How long been trying to quit**							
	More than a year	1		1		2	
	6 months to 1 year	2		1		3	
	Less than 6 months	3		4		7	
**Number of quit attempts**							
	More than 3	5		3		8	
	Less than 3	1		2		3	
**Reasons for quitting**							
	Health	3		3		6	
	Money	2		1		3	
	Money and health	1		2		3	
**Preferred quit approach**							
	Cold turkey	3		1		4	
	Gradual reduction	3		3		6	
	Gradual reduction and nicotine replacement therapy	1		2		3	
**Ever used quit e-cigarette app**							
	Never	6		6		12	

^a^e-cigarette: electronic cigarette.

In addition to the closed questions, we asked 3 open-ended questions. First, we asked if they could tell us what they did on a day that they left to do something and forgot to bring their vape. Both women and men said that they did 1 of 3 main things: smoked a cigarette, went back to get their e-cigarette, or bought a new vape device altogether. Second, we asked about what their friends and family did to support their quit efforts. Both women and men said that they received encouragement from family and friends, but women were more likely to report unhelpful support from family and friends, including telling them not to vape, and telling them that e-cigarettes are bad for them. Finally, we asked from whom they sought advice for quitting. While friends were the primary go to for both women and men, more men went to their family than women, and more women were likely to keep it to themselves compared with men.

### Desirability of an e-Cigarette Cessation App

All young adults unanimously agreed that reaching them through a smartphone app for supporting e-cigarette cessation aligned with their preferences for behavior change interventions. It aligned with their interest and proficiency in navigating new technologies:

I actually find the idea really quite smart. I think it's would definitely help like younger people who are on their phones a lot like I have a smartwatch, so I am already used to kind of depending on technology. So, I think that it would be really beneficial to the people who are like techno natives.

P12, male, 21

### Preferences for App Features and Content

#### Overview

Analysis of the data resulted in 4 categories related to app preferences: (1) flexible personalization (2) e-cigarette behavior tracking, (3) safely managed social support, and (4) positively framed notifications.

#### Flexible Personalization

Participants indicated that personalization was very important so that they felt supported in their individual quit journey. Key to this personalization was flexibility in terms of how they engaged with the app. At the outset, participants thought that having the option to sign-up through their email or various social media accounts (eg, Facebook) was the best way to begin use of the app:

Some people are sticklers for logging in with their other social media accounts. So, the ability to log in through any platform is definitely, it has to be there....why close the door on somebody ready to start?

P1, male, 27

Once they were signed up, participants reported that it was important for them to tailor the app according to their needs. As such, they wanted the app to have an option wherein they can input their e-cigarette behavior including amount consumed per day, level of addiction, situations of temptations, reasons for quitting, and personal goals:

I think it's important for a person who wants to quit e-cigarettes to give them the best chance maybe the software has to determine what level of addiction they have. So, you know, inputting the user's typical day and amount they smoke and then you know, calculating a reduction rate and maybe you know the ability to calculate when this person has the most usage maybe, you know asking questions like do you smoke at work? Do you work a stressful job? When do you smoke most?

P1, male, 27

Furthermore, to make the app more personalized and stand out from the rest of general intervention apps, they wanted the ability to set and modify their personalized goals.

I can personalize my stats, and my goals will motivate me to keep using the app.

P5, male, 26

Women were particularly insistent that the personalized content was matched with a personal look and feel. When they were talking about the design of the app, female participants suggested adding options to change the appearance of the app, such as changing the background color or adding pictures.

I love color. So, to be able to customize background colors and appearance of your dashboard, that would probably be really cool.....[I] would be more willing to, you know, log in every day and actually give it a shot.

P6, female, 20

They also mentioned the desire to have the current day/time portrayed, and a personalized greeting to be displayed when users log in the app.

When user logs in, it can say, like, good morning and then your name….to make it more personalized and to make it feel like the app is talking to you.

P10, female, 24

#### e-Cigarette Behavior Tracking

Another feature that the participants wanted in the app was to track their e-cigarette behavior, particularly their progress in staying away from using an e-cigarette and the direct benefits they could expect from this reduction in e-cigarette use. They wanted information about associated improvements in health and money saved listed as the top 2 items that they would like to be tracked, which directly reflected their top reasons for quitting. Additionally, participants wanted apps to have options to track withdrawal symptoms, progress to desired goals, and situations where they vape the most. The majority of participants wanted a graphical representation (pictures, charts, graphs, etc.) of the data monitored by the tracking feature to make it easier to understand:

Like [show] a picture of lungs, and then as the app goes on, and based on your own personal progress, like [show] the change in lungs, that's kind of cool.

P4, female, 26

#### Safely Managed Social Support

To make the app engaging, participants expressed a desire to see a social support or a community feature incorporated in the app. The participants indicated this feature would allow users to view and share their e-cigarette cessation journey, including any difficulties they are facing, as well as progress, making the process of quitting “less lonely.” One participant explained:

Like [part of the] recovery process is to have some camaraderie with other people that are going through the same thing whether it be drugs or alcohol or smoking. I definitely think that sense of community is really super important.

P11, female, 29

A few participants wanted a buddy option added with the community feature to enable others to provide encouragement, increase accountability, and to help them stay motivated. The use of tags was also suggested by participants, such as withdrawal symptoms, tips on setting goals, testimonies/advice from successful quitters, and inspirational messages/positive reinforcement. With these tags, users would have tools to navigate the community and find the support they needed.

Underpinning the desire for social support through an app, participants were adamant that the delivery platform is managed safely. Some suggested that a moderator in the community feature would be needed to prevent users from posting abusive and inappropriate content. Similarly, an age limit added to this feature was suggested to help make the community feature safe from predators.

[It] should have a moderator or an admin type so that it does not go out of hands and everyone stays civil.

P8, female, 27

Finally, participants showed their interest in sharing posts anonymously to ensure the privacy of app users:

A lot of people don't want to talk about why they're quitting. So having it be a bit more anonymous or being able to post anonymously.

P12, male, 21

[I would be] more comfortable using it if there was an anonymous posting feature, or like, it didn't include your name and your profile picture. And you could just choose the option to post anonymously.

P6, female, 20

#### Positively Framed Notifications

Participants suggested that an effective app needs to provide positively framed notifications that consist of motivational messages, encouraging reminders related to personal goals to strengthen and sustain motivation, and notifications that focus more on the benefits of quitting e-cigarettes rather than providing information on harmful effects of e-cigarettes.

Notification messages like, congratulations or like, keep going, you're doing great, that type of thing to encourage people. People respond well to acknowledgement, and like instant gratification, positive reinforcement.

P4, female, 29

Participants also wanted notifications to give insight into their progress, such as through an end of the day or weekly report:

You've gone 24 hours this week without an e-cigarette you know, you've cumulatively given up e-cigarettes for 48 hours, like those kind of milestones. So, I definitely think like those kind of milestone notifications might be helpful.

P11, female, 29

Additionally, participants expressed their interest in receiving updates and notifications on e-cigarette research and cessation from reliable sources.

## Discussion

### Principal Findings

In this study, we examined young adults’ perceptions and preferences around receiving e-cigarette cessation support via smartphone technology. The findings reveal that young adults not only want support via smartphone apps, but also that they want this support to be as tailored and as flexible as possible to meet their personal needs. Prior research on app-based health interventions indicates that users prefer customizable features that meet their personalized needs [24-26]. Unfortunately, however, a recent evaluation of health-promoting apps revealed that most apps incorporate a static, one-size-fits-all approach when it comes to personalization [26]. This is likely contributing to the ongoing finding that most app-based interventions have a minimal effect compared with usual care [26], including cessation apps [27]. Enhancing personalization of these interventions would arguably enhance the effect of these interventions. In relation to e-cigarettes, capturing granular information from an end user would ensure that the app learns about the characteristics of the vape user, and uses this information to tailor the type and intensity of intervention and target different aspects of the quitting process on an individual level. Future research on harnessing end user data for improved personalization and the impact it has on outcomes is needed.

We also found that the participants welcomed receiving positive, nonjudgmental notifications and messages versus receiving messages around the negative impacts of e-cigarette during their quit attempt through an app. This finding is in line with previous research findings indicating that young adults who are ready to quit prefer positively framed messaging to support smoking cessation [28,29]. In addition, positive message framing has been found to be more persuasive in prompting smoking cessation compared with negative message framing [30]. However, one must be cautious when considering these findings in light of evidence that message framing should be considered in the context of the cessation trajectory [31], nicotine-dependence levels [32], and user characteristics, such as gender [33]. For example, Cornacchione and Smith [31] found that smokers were more receptive to positive message framing if they were moving from contemplation to preparation to quit. In this regard, positive message framing may be most effective and helpful when a user first signs up and during the early stages of quitting, with negative messaging an effective approach later on once their confidence and motivation are higher (eg, present what they would lose if they started e-cigarettes again). Future research on the preferences of young adults moving through the process of e-cigarette cessation is needed to determine the most effective messaging at different time points for different end users.

Tracking one’s behavioral patterns was perceived as a critical component in an e-cigarette cessation app. To track user’s behaviors and outcomes more precisely, designers should consider designing smart cases for e-cigarette products that could collect e-cigarette intake and related data and send them to the smartphone app for advanced analysis [34,35]. This could complement the mobile app–based e-cigarette cessation intervention. The VapeTracker, which tracks the number of puffs and puff duration by attaching it to an e-cigarette device, is an example of an external device that vapers perceived positively [35]. Exploring user preferences around how an external device could be engaged to support their e-cigarette cessation needs further study.

This study also indicated a difference in the preference of young men and women in the design of the app, with female participants wanting more tailoring in terms of the appearance of the app (such as background color, font size/color, greetings from the app when logged in) compared with male participants. In addition, according to the demographic questionnaire data, female participants often used e-cigarettes to cope with stress, which holds implications for incorporating stress management strategies (eg, meditation, exercise) into the app. We also observed that male participants preferred quitting cold turkey, whereas women preferred a more gradual approach toward quitting. This is similar to findings from research related to preferences for a smoking cessation app, where young men reported a preference for cold turkey and young women preferred a gradual approach to quitting [36]. It has been suggested that these preferences may tie into heteronormative narratives (eg, men can quit when they want; women are more open to receiving help), and that masculinities (eg, autonomy and ability) and femininities (eg, caring and emotionally attuned) carry potential in being leveraged to support behavior change [36]. These noted differences in preferences suggest that attention to health equity and gender influences is necessary when developing such interventions.

### Comparison With Other Work

When examining other app-based interventions, only 3 were found in the iTunes and Apple stores (Quit e-Cigarettes, Escape the Vape, and Quit E-cigarette Addiction) that specifically targeted e-cigarettes (versus smoking or smoking and e-cigarette). None of these apps have been evaluated in the scientific literature to determine the level of personalization, social support, tracking features, and type of messaging. The text-messaging intervention (This is Quitting) [37] to help young people quit e-cigarettes has been evaluated. This program offers young people a minimum of 4 weeks of messages tailored to age and a resettable quit date, which are focused on skills and confidence building [37]. Results from a randomized control trial revealed that this program achieved 30-day point prevalence abstinence of 24.1% at 7 months compared with the control (18.6%) [12], adding support to our findings in relation to the value of personalization and positive-message framing. Our findings also suggest that there is an appeal among users for gamification and behavioral self-monitoring when it comes to e-cigarette interventions. Therefore, future researchers should consider how incorporating such features into a potential app might increase the acceptability of this type of cessation intervention, and explore the longevity of app-based e-cigarette cessation.

Researchers have indicated that there is an urgent need for research to inform vaping cessation programs for young adults and enhance an evidence base [38]. While the findings of our study fill some important content-related gaps, more research is needed to understand how a cessation app might account for the context of young adult vaping, including varying patterns of use, co-use with other products (eg, tobacco and cannabis), various perspectives and experiences with quitting, and various motives for both vaping and cessation [38]. Future research, therefore, not only needs to test features described in this study, but also needs to pay attention to the complexity of young adult vaping in order to successfully advance science in this area.

### Limitations and Strengths

A limitation of this study is that it was conducted with 12 participants, and there may be additional views that are not represented in this sample. Further, given our recruitment procedures (online and through a university), we acknowledge that our findings are limited to a sample that has internet access and is likely pursuing a postsecondary education. We are, however, confident that the study provides important insights that can be used as a foundation for guiding the development of novel apps to support e-cigarette cessation. In addition, our findings do not account for new regulatory policies that may impact the way people vape and their needs for support (eg, policies around nicotine concentration, where you can vape, taxation on e-cigarettes). Therefore, our study does not account for the influence of a wide variety of e-cigarette policies and its impact on e-cigarette use. As such, an app would need to be attuned to those policies. Strengths of this study include equal representation of young women and men, providing a more equitable look at what end users would want from a cessation app. Another strength of the study is the use of inductive qualitative methods to identify key themes in relation to app features. This lends to a strong evidence base from which to move forward with recommendations.

### Conclusion

In this qualitative study, we took an initial step to explore the preferences and perceptions of young adults on mobile app–based e-cigarette cessation interventions. Participants provided suggestions on the content and design of the mobile app–based intervention for e-cigarette cessation. These suggestions provide direction for the development and testing of technology-based e-cigarette cessation interventions for young adults.

## Appendix

Multimedia Appendix 1 Interview questions.

## Abbreviations

e-cigarettes: electronic cigarettes
