# Supplementary material for: Preferences for Mobile-Supported e-Cigarette Cessation Interventions Among Young Adults: Qualitative Descriptive Study
Source: JMIR Form Res. 2022 Apr 1;6(4):e33640. doi: 10.2196/33640 (PMC9015737; doi:10.2196/33640)
Supplement: Multimedia Appendix 1 [file formative_v6i4e33640_app1.docx]

| **Interview guide topic** | **Interview questions** |
| --- | --- |
| ***App appeal*** | What do you think of having an app available to support quitting vaping? |
| ***Log in and sign up*** | How do you want to log into the app (e.g., email, phone number)? |
|  | How important are personalization questions (e.g., puffs per day)? |
|  | What other types of personalization would you want (e.g., look and feel)? |
| ***Notifications*** | What types of notifications would you like to receive? |
|  | How would you like to receive notifications (e.g., frequency)? |
| ***Goals feature*** | Would you want to list your goals for being vape free? |
|  | What types of goals would you want incorporated into the app? |
| ***Tracking feature*** | What do you think of a feature that tracks your progress (e.g., money saved)? |
|  | What would you want to track in the app? |
| ***Community feature*** | What do you think of having a community feature for app users? |
|  | How would you want the community set-up (e.g., anonymous)? |
| ***Overall feedback*** | What other suggestions do you have for app features? |
|  | What advice would you have for an app developer? |
